# Genetic variants in the adenosine triphosphate-binding cassette transporter A1 and risk of age-related macular degeneration

**DOI:** 10.1007/s10654-023-01021-4

**Published:** 2023-06-19

**Authors:** Liv Tybjærg Nordestgaard, Mette Christoffersen, Shoaib Afzal, Børge Grønne Nordestgaard, Anne Tybjærg-Hansen, Ruth Frikke-Schmidt

**Affiliations:** 1 Department of Clinical Biochemistry, Copenhagen University Hospital - Rigshospitalet, Copenhagen, Denmark; 2 Department of Clinical Biochemistry, Copenhagen University Hospital - Herlev and Gentofte, Copenhagen, Denmark; 3 Department of Clinical Medicine, Faculty of Health and Medical Sciences, University of Copenhagen, Copenhagen, Denmark; 4 The Copenhagen General Population Study, Department of Clinical Biochemistry, Copenhagen University Hospital - Herlev and Gentofte, Copenhagen, Denmark; 5 The Copenhagen City Heart Study, Copenhagen University Hospital - Bispebjerg and Frederiksberg, Copenhagen, Denmark

**Keywords:** High-density lipoprotein, Drusen, ATP-binding cassette transporter A1, Blindness, Cholesterol, Genetics

## Abstract

Genetic variants in *ABCA1* are associated with higher concentrations of high-density lipoprotein (HDL) cholesterol. Higher HDL cholesterol concentrations are observationally and genetically associated with higher risk of age-related macular degeneration (AMD). However, whether amino acid-changing genetic variants in *ABCA1* associated with high HDL cholesterol concentrations confer a higher risk of AMD in the general population is currently unknown. We tested this hypothesis. The study included 80,972 individuals (1,370 AMD cases) from the Copenhagen General Population Study (CGPS) and 9,584 individuals (142 AMD cases) from the Copenhagen City Heart Study (CCHS) with 10 to 18 years of follow-up. We created an HDL cholesterol weighted allele score based on amino acid-changing *ABCA1* variants with a minor allele frequency above 0.001 and divided it into tertiles. The study included 55% women. Mean age was 58 years. The *ABCA1* allele score for the third versus the first tertile was associated with HRs (95% confidence intervals (CIs)) of 1.30 (1.14–1.49) for all-cause AMD, 1.26 (1.06–1.50) for nonneovascular AMD, and 1.31 (1.12–1.53) for neovascular AMD in a multivariable adjusted model. On a continuous scale, higher concentrations of genetically determined HDL cholesterol were associated with higher risk of all-cause AMD, nonneovascular AMD, and neovascular AMD in an age- and sex adjusted model and in a multivariable adjusted model. In conclusion, amino acid-changing genetic variants in *ABCA1* associated with higher HDL cholesterol concentrations were also associated with higher risk of AMD, suggesting a role for ABCA1 in AMD pathogenesis.

**Supplementary Information:**

The online version contains supplementary material available at 10.1007/s10654-023-01021-4.

Age-related macular degeneration (AMD) is a disease of the retina characterized by lipid-rich deposits called drusen which accumulate beneath the basal lamina of the retinal pigment epithelium (RPE)[1], causing progressive loss of central vision[2]. Clinically, it is subdivided into early stage AMD and late stage AMD[2], and late stage can be further subdivided into nonneovascular and neovascular AMD[2]. It is a leading cause of blindness affecting approximately 8.4 million individuals worldwide, especially in regions of the world with high proportions of elderly people where up to 14% of blindness cases are caused by the disease[3]. However, other than age, studies have shown that genetic risk factors contribute significantly to risk of AMD[4].

Genetic risk factors for AMD include genes involved in lipid metabolism including the cholesteryl ester transfer protein gene *(CETP)*[5], the hepatic lipase gene *(LIPC)*, the apolipoprotein E gene *(APOE)*, and the adenosine triphosphate-binding cassette transporter A1 (*ABCA1*) gene [4, 6]. These genes are all involved in the metabolism of high-density lipoprotein (HDL) cholesterol, however, the exact mechanism underlying the involvement of these genes in the pathogenesis of AMD remains unknown. ABCA1 is a cholesterol transporter that transports cholesterol onto lipid-poor apolipoproteins[7, 8] and is expressed in various tissues including the eye, brain, and liver. Homozygotes for loss-of-function mutations in *ABCA1* have Tangier disease, a disease characterized by extremely low plasma concentrations of HDL cholesterol and accumulation of cholesterol in the tonsils, liver, spleen, lymph nodes, and cornea[9]. Intronic variants in *ABCA1* are reported to be associated with risk of AMD in genome-wide association studies (GWAS)[4, 6], but whether this also applies to amino acid-changing *ABCA1* variants in the general population is unknown.

Using two large population cohorts including 90,556 individuals, we tested the hypothesis that amino acid-changing genetic variants in *ABCA1* associated with high HDL cholesterol concentrations are also associated with risk of AMD in the general population.

## Methods

### Setting, study population, and outcome

We included 90,556 individuals from two similar prospective cohort studies of the Danish general population, the Copenhagen City Heart Study (CCHS, n = 9,584) and the Copenhagen General Population Study (CGPS, n = 80,972). We excluded individuals with prevalent AMD (n = 50) at baseline. Institutional Review Board and Ethics Committee approval was obtained (KF-100-2039/91 and H-KF-01-144/01) and the study was conducted according to the Declaration of Helsinki. Written informed consent was obtained from all participants. All participants were White and of Danish descent. For an overview of the study design see Supplementary Fig. 1.

#### CGPS

The CGPS was initiated in 2003 with ongoing enrolment. Individuals were selected based on the national Danish Civil Registration System to reflect the adult Danish population aged 20–100 years or older. Data were obtained from a questionnaire, a physical examination, and a blood sample. We included 80,972 consecutive participants from this study in the present analysis. A total number of 1,370 AMD patients were diagnosed by the end of follow-up in the CGPS. Median follow-up was 10 years (< 1 to 15 years).

#### CCHS

The CCHS was initiated in 1976-78, with follow-up examinations in 1981-83, 1991-94, and 2001-03. Participants were recruited and examined as in the CGPS. We included all 9,584 consecutive participants in the 1991-94 and 2001-03 examinations in the present analysis. A total number of 142 AMD patients were diagnosed by the end of follow-up in the CCHS. Median follow-up was 18 years (< 1 to 27 years).

#### Endpoint definition

A diagnosis of AMD (World Health Organization, *International Classification of Diseases*, *Tenth Revision* [ICD-10]: DH353C, DH353E, DH353J, DH353K, DH353L, and DH353M, including senile macular degeneration (atrophic or exudative, with and without subretinal neovascularization and/or vitreomacular traction) was ascertained using the national Danish Patient Registry, which registers all contacts and diagnoses from public and private hospitals, from study entry until December 18th, 2018; not a single person was lost to follow-up due to the unique Danish registries. Danish national guidelines direct private practicing ophthalmologists to refer patients suspected of having exudative or neovascular AMD with active choroidal neovascularization to anti-VEGF treatment centralized at hospitals. This secures that most patients with AMD are registered with a diagnosis at a hospital, while all diagnoses of AMD in Denmark are given by medical doctors specialized in ophthalmology. The referral decision is (as a minimum) based on (1) visual acuity measurement, (2) slit lamp examination, ophthalmoscopy, or color fundus photography after pupil dilation, and (3) optical coherence tomography scan. Neovascular AMD was ICD10: DH353C, DH353J, DH353K, and DH353M. Nonneovascular AMD was ICD10: DH353E and DH353L. Individuals diagnosed with both nonneovascular and neovascular AMD were included in analyses of both endpoints. There were 391 individuals in CGPS and 35 individuals in CCHS who were diagnosed with both nonneovascular and neovascular AMD. The diagnoses of skin cancer were classified according to the World Health Organization international classification of diseases 10th edition (ICD-10): non-melanoma skin cancer as C44 and melanoma as C43. None of the non-melanoma skin cancer or melanoma diagnoses were based on self-report, and all were from the Danish Cancer Registry.

### Genotyping

All 90,556 individuals were genotyped for fourteen amino acid-changing variants in *ABCA1*: rs145183203, rs2230806, rs9282543, rs2066718, rs35819696, rs138880920, rs2066715, rs2066714, rs1422877738, rs33918808, rs76881554, rs2230808, rs146292819, and rs142688906. Briefly, these fourteen variants were selected because they were amino acid-changing and had a minor allele frequency above 0.001. From these fourteen variants nine were chosen for further analyses because of a significant association with HDL cholesterol concentrations in plasma (rs145183203, rs2066718, rs35819696, rs2066715, rs2066714, rs33918808, rs76881554, rs2230808, rs146292819). Genotyping was by TaqMan-based assays (Applied Biosystems, Foster City, CA, USA), or by an allele-specific PCR (Fluidigm; BioXpedia, Aarhus, Denmark). See Supplementary Fig. 2 for an overview of the genotyping and selection of variants for further analyses. Rs429358 and rs7412 in *APOE*, rs708272 in *CETP*, and rs1800588 in *LIPC* were genotyped as previously described[5, 10, 11].

### Other covariates

Covariates for baseline characteristics were risk factors for atherosclerotic cardiovascular disease and AMD: age, sex, smoking status, alcohol consumption, use of lipid-lowering therapy, diabetes, hypertension, body mass index, physical inactivity, education, postmenopausal status, and hormonal replacement therapy. These risk factors were chosen since they have all been shown to be associated with risk of AMD[2, 12–16]. Hypertension was use of anti-hypertensive medication and/or a systolic blood pressure of ≥ 140 mmHg, and/or a diastolic blood pressure of ≥ 90 mmHg. The reference was a systolic blood pressure < 140 mmHg or a diastolic blood pressure < 90 mmHg. Diabetes mellitus was self-reported disease, use of insulin or oral hypoglycaemic agents, and/or non-fasting plasma glucose levels of > 11 mmol/L (198 mg/dL). The reference was no self-reported disease or non-fasting plasma glucose levels ≤ 11 mmol/L (198 mg/dL). Smoking was current smoking. The reference was non-smokers. High alcohol consumption was > 14/21 units per week for women/men (1 unit = 12 g alcohol, equivalent to one glass of wine, one shot of spirit, or one beer (33 cL)). The reference was ≤ 14/21 units per week for women/men. Physical inactivity was ≤ four hours per week of light physical activity in leisure time. The reference was > four hours per week of light physical activity in leisure time. Women reported menopausal status (the reference was premenopausal women) and use of hormonal replacement therapy (in women who were postmenopausal). The reference was no use of hormonal replacement therapy (in women who were postmenopausal). Lipid-lowering therapy was primarily statins (yes/no). The reference was no use of lipid-lowering therapy.

### Statistical analyses

R version 3.6.1 or Stata SE 17 was used. Chi-squared tests evaluated Hardy-Weinberg equilibrium. Kruskal-Wallis test or Cuzick’s test for trend was used to compare continuous covariates and Pearson’s chi-squared test to compare categorical covariates by genotype or weighted allele score. Missing data on covariates (0.4%) were imputed based on all other covariates in the study and their distributions using multivariable linear regression for continuous variables. Categorical variables where assigned a separate category. The mi impute command in Stata was used. If only individuals with complete data were included (no use of imputed data), results were like those reported.

In genetic analyses, we defined the exposure allele for each of the nine variants as the allele associated with the highest HDL cholesterol. For an in-depth description of the construction of the genetic score see Supplementary Table 1. For each individual, the nine variants were combined in an allele score weighted on higher HDL cholesterol (Supplementary Table 1) by multiplying the adjusted β-coefficient (see below) for each variant by the frequency of that variant and adding the adjusted β-coefficients across all variants. The adjusted β-coefficient for a specific variant takes the linkage structure from all nine variants into account by adjusting for the effects of all other variants in a linear regression analysis. The weighted allele scores across all nine variants for the different combined genotypes observed were then divided into groups of approximately equal size (called tertiles in the following): adjusted betascore from − 0.44 to < 0 (first tertile = reference), 0 (second tertile), and from > 0 to 0.13 (third tertile). External weights from https://app.genebass.org/ were used to create a weighted allele score in a sensitivity analysis (Supplementary Table 1).

To examine the possible causal association between lifelong amino acid-changing genetic variants in *ABCA1* and risk of AMD we used Cox proportional hazard models with age as time scale and delayed entry at examination (left truncation). For Cox regression models, proportionality of hazards over time was assessed by plotting -ln(-ln[survival]) versus ln(analysis time). There was no suspicion of non-proportionality. All models were age- and sex adjusted and multivariable adjusted for risk factors for cardiovascular disease (other than lipids) and AMD: age (as time scale), sex, cohort, date of birth, body mass index, hypertension, diabetes, physical activity, smoking, alcohol consumption, postmenopausal status (in women), use of hormone replacement therapy (in women), education, and use of lipid-lowering therapy. In sensitivity analyses we further adjusted for *APOE, CETP*, and *LIPC* genotype, for LDL- and total cholesterol, as well as for skin cancer as a proxy for extensive sun exposure. The association between the *ABCA1* weighted allele score and risk of AMD was examined on a continuous scale using restricted cubic splines incorporated in the Cox regression model. The number of knots was chosen according to Akaike information criteria[17]. The reference was set to a weighted allele score of 0 to have the largest possible reference group (Supplementary Table 1, panel B).

Interactions were tested using an interaction term between the *ABCA1* weighted allele score and covariates, one at a time, in a multivariable adjusted Cox model. When investigating the interaction between age and the *ABCA1* weighted allele score on AMD, time since study entry was used as the time scale.

In mediation analyses, the percent of the total risk of AMD conferred by genetic variants in *ABCA1* which was associated with genetically higher HDL cholesterol, was determined by the Karlson-Holm-Breen method[18], using bootstrapping to calculate confidence intervals.

Associations of plasma HDL cholesterol (mmol/L) with LDL- and total cholesterol concentrations (mmol/L) were investigated using multiple linear regression and graphically displayed using kernel-weighted local polynomial smoothing and geometric means with 95% confidence intervals (CIs).

## Results

Baseline characteristics of study participants by tertiles of *ABCA1* allele score weighted on HDL cholesterol are shown in Table 1. Mean (standard error) HDL cholesterol was 1.59 mmol/L (0.50) in the first tertile (reference), 1.62 mmol/L (0.51) in the second tertile (1.8% higher), and 1.65 mmol/L (0.53) in the third tertile (3.6% higher) of the *ABCA1* allele score (Supplementary Fig. 3). After allowing for 12 multiple comparisons (required p-value 0.05/12 = 0.004), none of the potential confounders were associated with the *ABCA1* allele score. There was no interaction between cohort and the weighted allele score in predicting risk of AMD (P for interaction = 0.49). Consequently, analyses were performed on the cohorts combined throughout the paper.

**Table 1:** Characteristics of study participants by groups of HDL cholesterol weighted allele score tertile

Characteristic	Weigthed allele score, No. (%)^a^			
	First tertile (n = 28,376)	Second tertile (n = 38,269)	Third tertile (n = 23,911)	P value^b^
HDL cholesterol, mean (SE),				
mmol/L	1.59 (0.50)	1.62 (0.51)	1.65 (0.53)	< 0.001
mg/dL	61 (19)	63 (20)	64 (20)	< 0.001
Age, mean (SE), y	57.8 (0.1)	57.9 (0.1)	57.5 (0.1)	0.03
Women	15,657 (55)	20,890 (55)	13,109 (55)	0.33
Body mass index^c^, mean (SE)	26.2 (0.0)	26.1 (0.0)	26.1 (0.0)	0.54
Hypertension^d^	16,068 (57)	21,684 (57)	13,543 (57)	> 0.99
Diabetes mellitus^e^	1663 (6)	2172 (6)	1416 (6)	0.39
Smoking ^f^	5645 (20)	7604 (20)	4778 (20)	> 0.99
High alcohol consumption^g^	4550 (16)	6313 (17)	3939 (17)	0.51
Physical inactivity^h^	14,040 (50)	18,857 (50)	11,627 (51)	0.30
Postmenopausal^i^	10,585 (68)	14,221 (68)	8809 (67)	0.26
Hormonal replacement therapy^i^	1699 (11)	2294 (11)	1443 (11)	0.46
Lipid-lowering therapy^j^	3095 (11)	4167 (11)	2692 (11)	0.67
Education < 8 years	3610 (13)	4709 (12)	2855 (12)	0.03

Abbreviation: HDL, high-density lipoprotein

^a^ Missing data on continuous covariates (< 0.5%) were imputed based on sex, age, hypertension, diabetes, smoking status, use of lipid-lowering therapy, alcohol consumption, education, physical inactivity, menopausal status, and use of hormonal replacement therapy

^b^*P* for differences are by *P* for trend (for body mass index), Kruskal-Wallis equality-of-populations rank test (for age), or by the Pearson Chi-squared test. The lowest tertile was used as reference

^c^ Calculated as weight in kilograms divided by height in meters squared

^d^ Hypertension was use of anti-hypertensive medication and/or a systolic blood pressure of 140 mmHg or higher, and/or a diastolic blood pressure of 90 mm Hg or higher

^e^ Diabetes mellitus was self-reported disease, use of insulin or oral hypoglycaemic agents, and/or non-fasting plasma glucose levels of more than 11 mmol/L (198 mg/dL)

^f^ Smoking was current smoking

^g^ High alcohol consumption was > 14/21 units per week for women/men (1 unit = 12 g alcohol, equivalent to one glass of wine, one shot of spirit, or one beer (33 cL))

^h^ Physical inactivity was ≤ four hours per week of light physical activity in leisure time

^i^ Assessed in women only. Women reported menopausal status and use of hormonal replacement therapy (in women who were postmenopausal)

^j^ Lipid-lowering therapy was primarily statins (yes/no)

### Risk of age-related macular degeneration as a function of *ABCA1* weighted allele score in tertiles

**Fig. 1:**
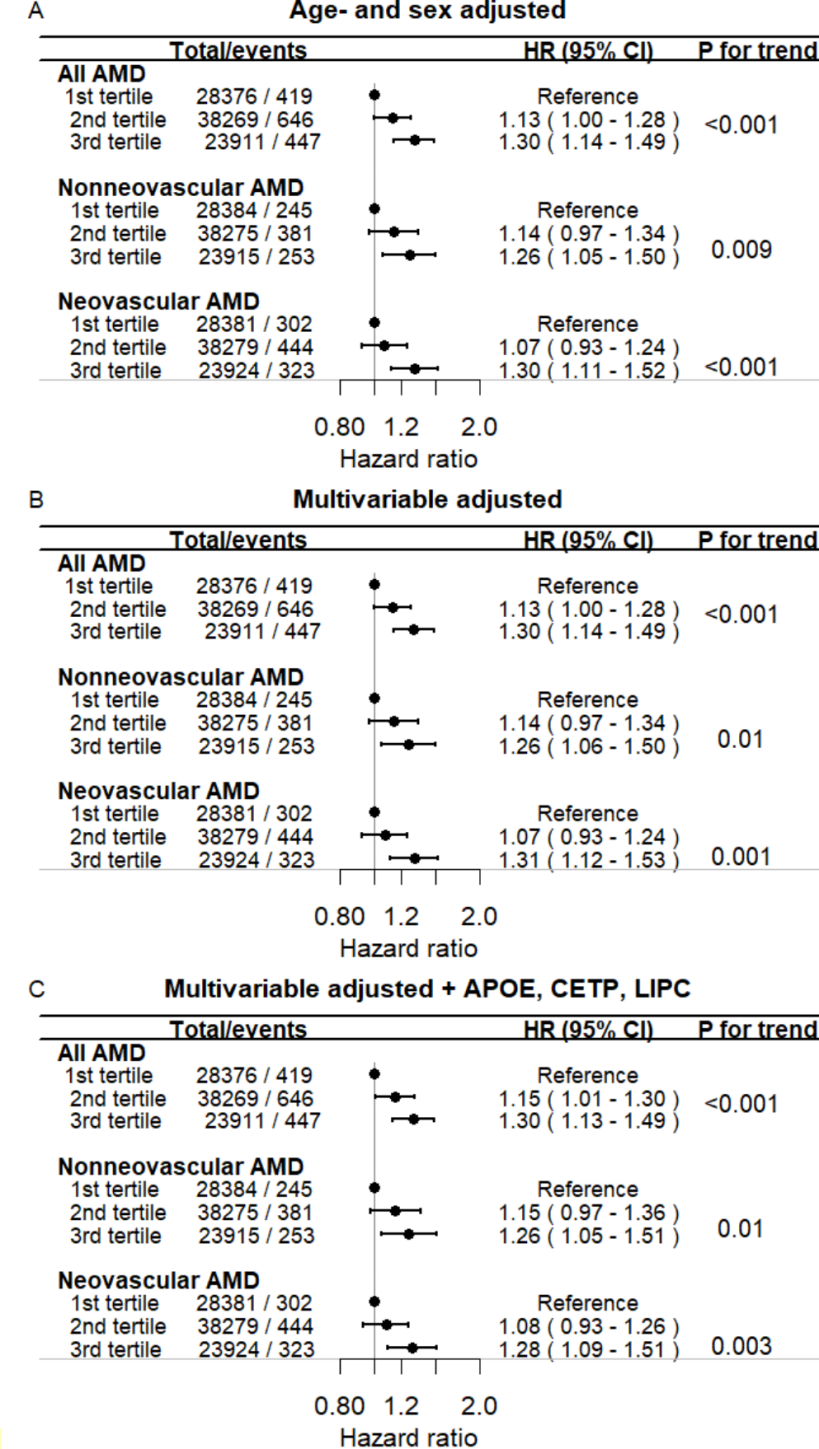
Risk of age-related macular degeneration as a function of *ABCA1* weighted allele score in tertiles. Hazard ratios (HRs) and 95% confidence intervals are from Cox regression models. The allele score was weighted on the effect on plasma HDL cholesterol and the allele frequency of the *ABCA1* amino acid-changing genetic variants in the CCHS and CGPS. Adjustment was for age, sex, and cohort (panel A), multivariable for age, sex, cohort, body mass index, hypertension, diabetes mellitus, smoking, alcohol consumption, physical inactivity, menopausal status, and hormonal replacement therapy (only women), lipid-lowering therapy, and education (panel B), and multivariable adjusted and further adjusted for *APOE, CETP*, and *LIPC* genotype (panel C). ABCA1 = adenosine triphosphate-binding cassette transporter A1; AMD = age-related macular degeneration; CCHS = Copenhagen City Heart Study; CGPS = Copenhagen General Population Study; CI = confidence interval; HDL = high-density lipoprotein

During a median follow-up of 10 years, 1,512 individuals developed all-cause AMD, 874 nonneovascular AMD, and 1,064 developed neovascular AMD. The multivariable adjusted hazard ratios (HRs) for all-cause AMD versus individuals in the first tertile of the *ABCA1* allele score were 1.13 (1.00 − 1.28) for those in the second and 1.30 (1.14 − 1.49) for individuals in the third tertile (Fig. 1, panel B top; P for trend < 0.001). The corresponding HRs for nonneovascular AMD were 1.14 (0.97 − 1.34) and 1.26 (1.06 − 1.50) (Fig. 1, panel B middle; P for trend = 0.01), while HRs for neovascular AMD were 1.07 (0.93 − 1.24) and 1.31 (1.12 − 1.53) (Fig. 1, panel B bottom, P for trend = 0.001), respectively. When further adjusting for *APOE, CETP*, and *LIPC* genotypes results were similar (Fig. 1, panel C). When using external weights results were also similar (Supplementary Fig. 4). Finally, results were similar when further adjusting for plasma LDL- and total cholesterol concentrations (Supplementary Fig. 5 and when further adjusting for skin cancer as a proxy for extensive sun exposure (Supplementary Fig. 6).

### Risk of age-related macular degeneration as a function of the *ABCA1* weighted allele score on a continuous scale

**Fig. 2:**
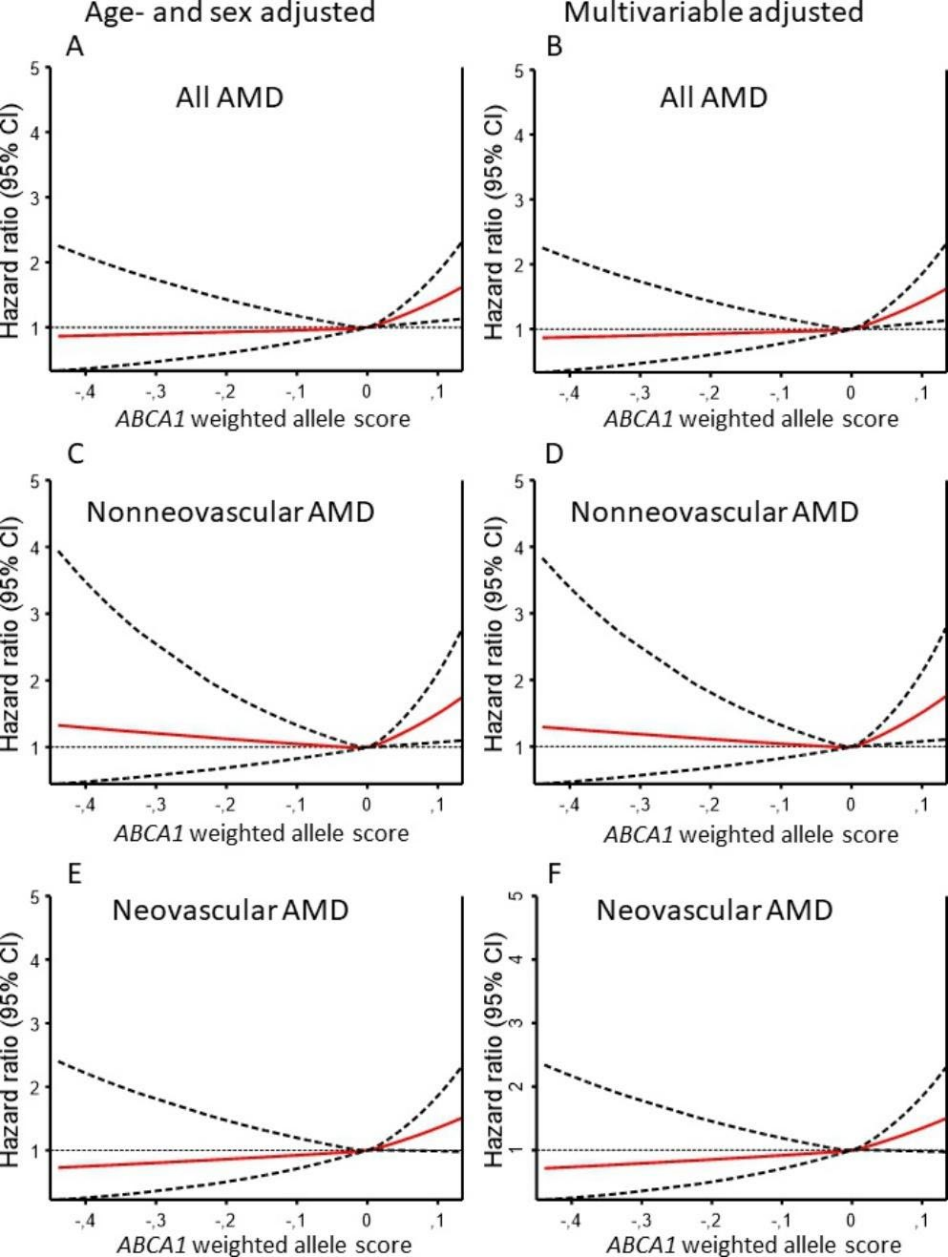
Risk of age-related macular degeneration as a function of the *ABCA1* weighted allele score on a continuous scale. Hazard ratios (red solid line) and 95% confidence intervals (thick, black dashed lines) are from Cox regression models using restricted cubic splines. The reference of 1.0 (thin dotted line) was set to a weighted allele score of 0 to have the largest possible reference group. Adjustment was for age, sex, and cohort (panel A, C, E) and multivariable for age, sex, cohort, body mass index, hypertension, diabetes mellitus, smoking, alcohol consumption, physical inactivity, menopausal status, and hormonal replacement therapy (only women), lipid-lowering therapy, and education (panel B, D, F). ABCA1 = adenosine triphosphate-binding cassette transporter A1; AMD = age-related macular degeneration; CI = confidence interval

On a continuous scale using restricted cubic splines, higher weighted allele score and thus higher genetically determined HDL cholesterol was associated with a higher risk of AMD, nonneovascular AMD, and neovascular AMD both in age- and sex adjusted and in multivariable adjusted models (Fig. 2).

### Risk of age-related macular degeneration per 1 higher *ABCA1* weighted allele score tertile in different strata of other risk factors

**Fig. 3:**
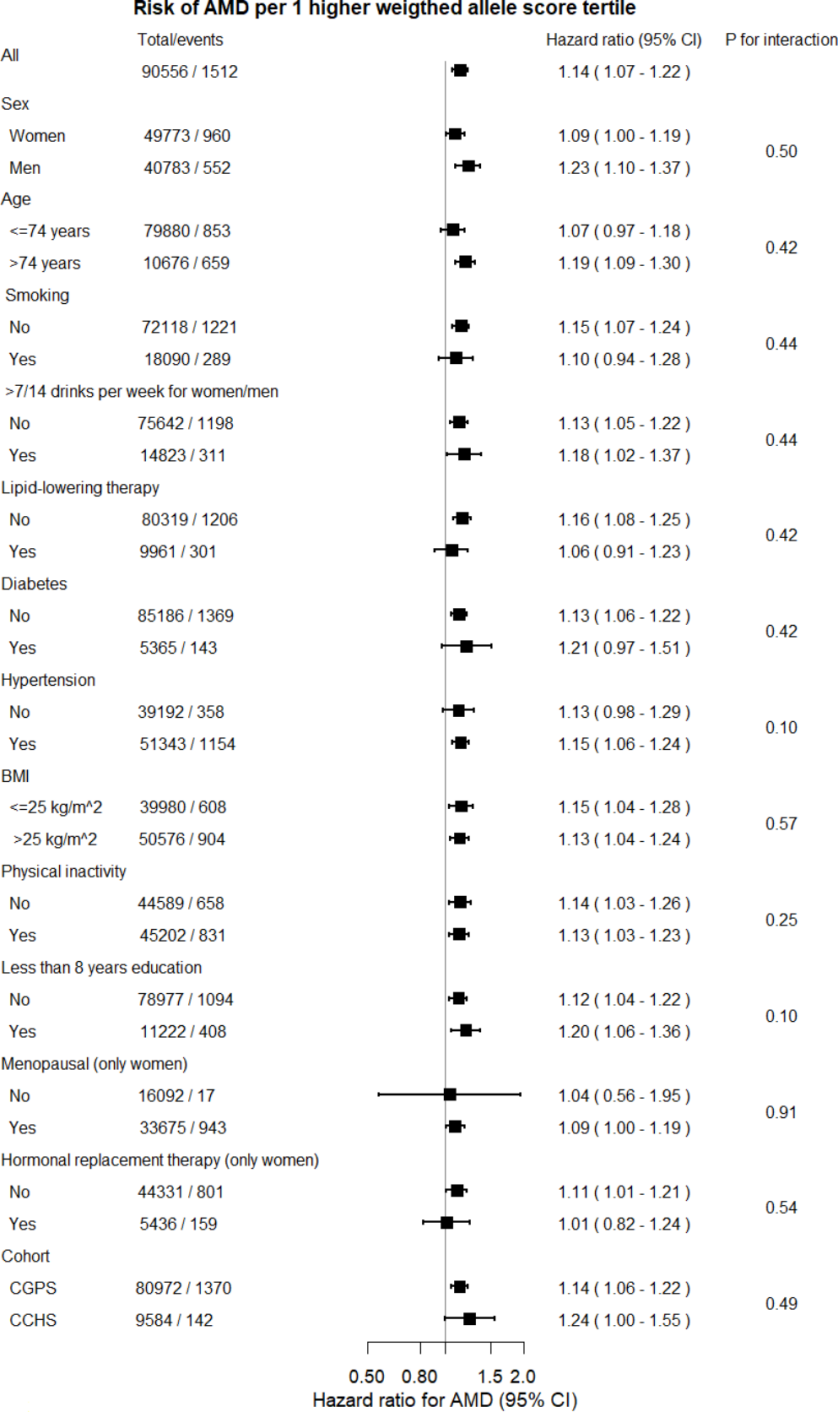
Risk of age-related macular degeneration per 1 higher *ABCA1* weighted allele score tertile in different strata of other risk factors. Hazard ratios (HRs) and 95% confidence intervals (CIs) were obtained from Cox proportional hazards regression. Age adjustment was through age as time scale, with no other adjustment. Number of total individuals and those with AMD events vary slightly for each stratum according to availability of data. ABCA1 = adenosine triphosphate-binding cassette transporter A1; AMD = age-related macular degeneration; BMI = body mass index. Hypertension was use of anti-hypertensive medication and/or a systolic blood pressure of 140 mmHg or higher, and/or a diastolic blood pressure of 90 mmHg or higher. Diabetes mellitus was self-reported disease, use of insulin or oral hypoglycaemic agents, and/or non-fasting plasma glucose levels of more than 11 mmol/L (198 mg/dL). Smoking was current smoking. High alcohol consumption was > 14/21 units per week for women/men (1 unit = 12 g alcohol, equivalent to one glass of wine, one shot of spirit, or one beer (33 cL)). Physical inactivity was ≤ four hours per week of light physical activity in leisure time. Women reported menopausal status and use of hormonal replacement therapy (in women who were postmenopausal). Lipid-lowering therapy was primarily statins (yes/no)

Risk of AMD was similar in different strata of other risk factors (all p-values for interaction between covariates and the weighted allele score on risk of AMD ≥ 0.10) (Fig. 3). As expected, a few 95% confidence intervals for the HRs overlapped 1.0 in some subgroups, including in those with the lowest number of individuals and AMD events, likely due to limited statistical power in these subgroups.

### Sensitivity analyses

#### Plasma concentrations of lipids, lipoproteins, and apolipoproteins

Associations of amino acid-changing genetic variants in *ABCA1* with plasma concentrations of lipids, lipoproteins, and apolipoproteins are shown in Supplementary Fig. 3 for tertiles of the weighted allele score based on the nine variants associated with HDL cholesterol and in Supplementary Figs. 7 and 8 for each of the 14 variants genotyped in CGPS and CCHS. Compared to individuals in the first tertile, HDL cholesterol was higher by 1.8% (1.2 mg/dL; 0.03 mmol/L) for those in the second tertile and by 3.6% (2.3 mg/dL; 0.06 mmol/L) for those in the third tertile (P for trend < 0.001); coefficient of determination was 0.2% while the F-value was 191. Corresponding values for apolipoprotein A1 were 1.3% (2 mg/dL) and 2.5% (4 mg/dL) (P for trend < 0.001), respectively, with coefficient of determination of 0.2% and an F-value of 153. The corresponding slight increase in plasma total cholesterol was explained by the HDL cholesterol increase, as LDL cholesterol, apolipoprotein B, and non-HDL cholesterol were unaffected by *ABCA1* allele score tertiles.

#### Single genetic variants

Single per allele weights used for calculation of the HDL weighted allele score are shown in Supplementary Table 1 A. Risk of all-cause AMD for each *ABCA1* variant is shown in Supplementary Fig. 8. These analyses highlight the contribution of each variant to the overall weighted allele score and to the overall risk of AMD. Results were compatible with the main results.

#### Mediation analyses

In mediation analyses genetic variants in *ABCA1* contributed with 6% (3–14%) of the increased risk of all AMD, 8% (3–30%) of the increased risk of nonneovascular AMD, and 6% (3–17%) of the increased risk of neovascular AMD via higher HDL cholesterol (Supplementary Table 2).

#### Correlation analyses

Higher plasma LDL cholesterol concentration was associated with lower plasma HDL cholesterol concentration with an r-squared of 0.17 (Supplementary Fig. 9). Plasma total cholesterol concentration was associated with a higher concentration of plasma HDL cholesterol concentration with an r-squared of 0.16 (Supplementary Fig. 9).

## Discussion

The principal findings are that amino acid-changing genetic variants in *ABCA1* which were associated with higher concentrations of HDL cholesterol, were associated with higher risk of AMD including both nonneovascular and neovascular AMD in the Danish general population. We observed these findings of genetically determined HDL cholesterol concentrations mediated by *ABCA1* both on a continuous scale and in categories. These findings are novel, because we only use amino acid-changing variants in *ABCA1*, variation that only affects HDL cholesterol concentrations and no other lipoproteins, thereby suggesting a likely causal relationship from *ABCA1* over HDL cholesterol to risk of AMD.

In support of our findings, the concentrations of various plasma lipids have been associated with risk of AMD, but the most robust association is observed between high plasma HDL cholesterol concentrations and higher risk of AMD in both observational and genetic analyses[5, 19–24], as also confirmed in the present study. Genes involved in HDL cholesterol metabolism that have also been associated with risk of AMD include *CETP, LIPC, APOE*, and *ABCA1*[5, 23]. The *ABCA1* gene is a significant signal in GWAS for AMD[4, 6], and in smaller studies, single variants in *ABCA1* are associated with risk of AMD[25] and progression from one stage of the disease to another[26]. While Mendelian randomization studies investigating the association between genetically determined HDL cholesterol concentration and risk of AMD have included *ABCA1* variants, no study has, to our knowledge, investigated the association between a weighted allele score based solely on amino acid-changing variants in *ABCA1* and risk of AMD[22–24]. This is important, as it may suggest direct causality from *ABCA1* over HDL to risk of AMD, and therefore highlights the possibility of using ABCA1 as a future drug target for preventing or maybe treating AMD.

The mechanism behind the association between genetically determined high concentrations of HDL cholesterol due to variation in *ABCA1* and risk of AMD is speculative, but below we describe a likely scenario. According to the “Oil spill hypothesis”, HDL particles are involved in the delivery of lipophilic nutrients to the retinal pigment epithelium (RPE) which is responsible for the subsequent delivery to the photoreceptors[1, 27]. The lipophilic nutrients are taken up by scavenger receptor class B type I (SR-BI) situated at Bruch’s membrane. Proteins involved in HDL metabolism are localized in the subretinal space and are thought to supply unesterified cholesterol to the retina. However, excess unesterified cholesterol is cytotoxic and must be offloaded from the RPE[1]. Mechanisms for offloading this excess unesterified cholesterol could include ABCA1-mediated transfer to circulating HDL particles[1] and to HDL particles within the RPE. The cholesterol content in these particles will be esterified, and over time clearance of lipoprotein particles across Bruch’s membrane is hindered by the accumulation of esterified cholesterol causing lipoproteins to be captured in Bruch’s membrane and forming a lipid wall external to the RPE basal lamina[1]. Subsequently, lipoproteins are degraded forming lipoprotein-derived debris. This eventually leads to the formation of the AMD-specific lesions called drusen[1]. Peroxidised lipids in drusen are thought to cause choroidal neovascularization through inflammation[1], which is characteristic of the type of AMD called “neovascular” AMD. Drusen will also lead to the blocking of transport of necessary nutrients from plasma to photoreceptors[1], and with time this will lead to neurodegeneration of photoreceptors and ultimately blindness characteristic of the type of AMD called “nonneovascular” AMD (Supplementary Fig. 10). Although speculative, it is thus biologically plausible that *ABCA1* variants associated with high HDL concentration may also be involved directly in the pathogenesis of AMD.

Strengths of the current study are the inclusion of a large homogeneous sample from the general population, the thorough characterization of risk factors for AMD on an individual participant level, and the prospective study design. Another strength is the adjustment for important risk factors for AMD and the additional adjustment for other important risk genes. Further, unlike previous studies, we investigated several amino acid-changing variants in *ABCA1* spanning the whole protein coding part of the gene.

Limitations include studying only White individuals of Danish descent; however, we are not aware of data suggesting that the present results should not apply to other ethnicities. Another limitation is the lack of functional studies of these structural genetic variants in *ABCA1*; however, the association with HDL cholesterol and apolipoprotein A1 concentrations in individuals in the general population is a reasonable proxy for functionality. It should be noted that there are other indicators of blood lipids besides HDL cholesterol. These include LDL cholesterol and total cholesterol that are only weakly correlated with HDL cholesterol. It is, however, unlikely that parts of the effect of *ABCA1* variants on risk of AMD are mediated through other lipids since the direct associations between *ABCA1* variants and other lipids and lipoproteins are negliable. Another limitation is that family history of AMD and known AMD loci as complement factor H, ARMS2, and VEGF are not included in the multivariable adjustment used throughout this study. Complement factor H, ARMS2, and VEGF are, however, encoded by genes located on other chromosomes than *ABCA1*, and are thus unlikely to bias the current results through linkage disequilibrium with *ABCA1* variants. Further, genetic results are very unlikely to be confounded[28] which is also clearly demonstrated by the similar results for three different adjustments in Fig. 1 and in Supplementary Figs. 5 and 6.

In conclusion, amino acid-changing genetic variants in *ABCA1* were associated with higher concentrations of plasma HDL cholesterol and higher risk of both nonneovascular and neovascular AMD in the Danish general population. These results may suggest causality from *ABCA1* over HDL cholesterol to risk of AMD and highlights the possibility of using ABCA1 as a future drug target for preventing or maybe treating AMD.

## Electronic supplementary material

Below is the link to the electronic supplementary material.

Supplementary Material 1

## Data Availability

The Danish data protection agency does not allow open access; however, upon reasonable request, additional analyses can be conducted after contact to the corresponding author.

## Abbreviations and acronyms

AMD: Age-related macular degeneration
ABCA1: Adenosine triphosphate-binding cassette transporter A1
BMI: Body mass index
CCHS: Copenhagen City Heart Study
CETP: Cholesteryl ester transfer protein
CGPS: Copenhagen General Population Study
APOE: Apolipoprotein E
HDL: High-density lipoprotein
HR: Hazard ratio
ICD: International Classification of Diseases
LIPC: Hepatic lipase
RPE: Retinal pigment epithelium
