# Supplementary material for: Genetic variants in the adenosine triphosphate-binding cassette transporter A1 and risk of age-related macular degeneration
Source: Eur J Epidemiol. 2023 Jun 19;38(9):985–94. doi: 10.1007/s10654-023-01021-4 (PMC10501952; doi:10.1007/s10654-023-01021-4)
Supplement: Supplementary file 1 — Supplementary Material 1 [file 10654_2023_1021_MOESM1_ESM.docx]

**Supplementary Information**

**Genetic variants in the adenosine triphosphate-binding cassette transporter A1 and risk of age-related macular degeneration**

Liv Tybjærg Nordestgaard MD, Mette Christoffersen MSc Pharm PhD, Shoaib Afzal MD DMSc, Børge Grønne Nordestgaard MD DMSc, DMSc, Anne Tybjærg-Hansen MD DMSc, Ruth Frikke-Schmidt MD

Journal name: European Journal of Epidemiology

Address for correspondence and reprints:

Ruth Frikke-Schmidt MD DMSc, Professor, Chief Physician

Department of Clinical Biochemistry, Section for External Projects

Rigshospitalet, Blegdamsvej 9, DK-2100 Copenhagen, Denmark,

Telephone: +4535454348; e-mail: [ruth.frikke-schmidt@regionh.dk](mailto:ruth.frikke-schmidt@regionh.dk)

**Contents**

[Supplementary Table 1 Single per allele weights (part A) used for high-density lipoprotein (HDL) weighted allele score calculation (part B). 3](#_Toc133136054)

[Supplementary Table 2 Explained risk in the association from genetic *ABCA1* variation based on 90,556 individuals from the Copenhagen General Population Study and the Copenhagen City Heart Study. 9](#_Toc133136055)

[Supplementary Figure 1 Study design 10](#_Toc133136056)

[Supplementary Figure 2 Genotyping of *ABCA1* amino acid changing variants in the CCHS and CGPS. 11](#_Toc133136057)

[Supplementary Figure 3 *ABCA1* weighted allele score in tertiles and lipid, lipoprotein, and apolipoprotein concentrations. 12](#_Toc133136058)

[Supplementary Figure 4 Risk of age-related macular degeneration as a function of *ABCA1* weighted allele score in tertiles using external weights 13](#_Toc133136059)

[Supplementary Figure 5 Risk of age-related macular degeneration as a function of *ABCA1* weighted allele score in tertiles multivariable adjusted including LDL and total cholesterol. 14](#_Toc133136060)

[Supplementary Figure 6 Risk of age-related macular degeneration as a function of *ABCA1* weighted allele score in tertiles multivariable adjusted including skin cancer. 15](#_Toc133136061)

[16](#_Toc133136062)

[Supplementary Figure 7 *ABCA1* variants and lipid, lipoprotein, and apolipoprotein levels. 17](#_Toc133136063)

[Supplementary Figure 8 Concentrations of HDL cholesterol and risk of AMD as a function of *ABCA1* genotype. 18](#_Toc133136064)

[Supplementary Figure 9 Correlation between plasma HDL cholesterol, LDL cholesterol, and total cholesterol 19](#_Toc133136065)

[20](#_Toc133136066)

[Supplementary Figure 10 The Oil Spill Hypothesis in Bruch’s membrane. 20](#_Toc133136067)

# Supplementary Table 1 Single per allele weights (part A) used for high-density lipoprotein (HDL) weighted allele score calculation (part B).

**A: Single per allele weights for nine amino acid-changing variants in the *ABCA1* gene on high-density lipoprotein (HDL) cholesterol.**

| **Chromosome position** | **Nucleotide substitution** | **Variant** | **Rs number** | **Number of individuals**  **(Minor allele frequency)** | | **Common allele** | **Minor allele** | **Single per allele weights** | **External weights** |
| --- | --- | --- | --- | --- | --- | --- | --- | --- | --- |
|  |  |  |  | **CCHS (9,584)** | **GGPS (80,972)** |  |  |  |  |
| 9:107646756 | c.254C>T | p.Pro85Leu | rs145183203 | 35(0.002) | 254(0.003) | C | T | -.2708796 | -.212 |
| 9:107589255 | c.2311G>A | p.Val771Met | rs2066718 | 642(0.03) | 5,323(0.03) | G | A | .1301543 | .104 |
| 9:107589246 | c.2320A>C | p.Thr774Pro | rs35819696 | 37(0.002) | 273(0.002) | A | C | -.13689 | -.0769 |
| 9:107588033 | c.2473G>A | p.Val825Ile | rs2066715 | 1,081(0.06) | 9,062(0.06) | G | A | .0526159 | .0597 |
| 9:107586753 | c.26494A>G | p.Ile883Met | rs2066714 | 2,166(0.12) | 18,094(0.12) | A | G | .0368484 | .0449 |
| 9:107579632 | c.3516G>C | p.Glu1172Asp | rs33918808 | 535(0.03) | 4,413(0.03) | G | C | .0523806 | NA |
| 9:107578620 | c.3542C>T | p.Ser1181Phe | rs76881554 | 74(0.004) | 635(0.004) | C | T | -.1436233 | -.205 |
| 9:107562804 | c.4760G>A | p.Lys1587Arg | rs2230808 | 3,998(0.24) | 34,306(0.24) | G | A | -.0379501 | -.0311 |
| 9:107556776 | c.5398A>C | p.Asn1800His | rs146292819 | 25(0.001) | 196(0.001) | A | C | -.8782658 | -.935 |

Variants with a frequency >15/10,000 in the CCHS were genotyped, of these nine had a significant effect on level of HDL-cholesterol. Number of individuals and rare allele frequencies in percent in brackets are shown. The single allele weights correspond to the per *ABCA1* allele β-coefficients adjusted for the impact of the other variants, derived from a linear regression in 90,556 individuals in the Copenhagen City Heart Study and the Copenhagen General Population Study including all nine *ABCA1* variants, age, sex, and cohort. Variants are annotated according to GRCh37/hg19, transcript NM_005502.3. External weights are from https://app.genebass.org/.

**B: Combined single allele weights into a plasma high-density lipoprotein weighted allele score.**

| **Pro85Leu** | **Val771Met** | **Thr774Pro** | **Val825Ile** | **Ile883Met** | **Glu1172Asp** | **Ser1181Phe** | **Lys1587arg** | **Asn1800His** | **Weights of allele combinations** | **Weighted allele score in 3 groups** | **Individuals** |
| --- | --- | --- | --- | --- | --- | --- | --- | --- | --- | --- | --- |
| CC | GG | AA | GG | AA | GG | CC | GA | AC | -0.43899 | 1 | 36 |
| CC | GG | AA | GG | AA | GG | CC | GG | AC | -0.4118 | 1 | 147 |
| CC | GG | AA | GA | AG | GG | CC | GA | AC | -0.40775 | 1 | 3 |
| CC | GG | AA | GA | AA | GG | CC | GG | AC | -0.399 | 1 | 1 |
| CC | GG | AA | GG | AA | GC | CC | GA | AC | -0.39488 | 1 | 5 |
| CC | GG | AA | GG | AG | GG | CC | GG | AC | -0.39336 | 1 | 6 |
| CC | GG | AA | GA | AG | GG | CC | GG | AC | -0.38056 | 1 | 11 |
| CC | GG | AA | GG | AG | GC | CC | GA | AC | -0.37644 | 1 | 2 |
| CC | GA | AA | GG | AA | GG | CC | GA | AC | -0.3678 | 1 | 3 |
| CC | GG | AA | GG | AA | GC | CC | GG | AC | -0.36769 | 1 | 1 |
| CC | GG | AA | GA | AG | GC | CC | GA | AC | -0.36365 | 1 | 1 |
| CC | GA | AA | GG | AA | GG | CC | GG | AC | -0.34061 | 1 | 4 |
| CC | GA | AA | GG | AA | GC | CC | GA | AC | -0.32369 | 1 | 1 |
| CT | GG | AC | GG | AA | GG | CC | GG | AA | -0.2245 | 1 | 1 |
| CT | GG | AA | GA | AG | GG | CT | GA | AA | -0.21861 | 1 | 1 |
| CT | GG | AA | GG | AA | GG | CC | GA | AA | -0.18302 | 1 | 52 |
| CT | GG | AA | GG | AG | GG | CC | GA | AA | -0.16458 | 1 | 5 |
| CT | GG | AA | GG | AA | GG | CC | GG | AA | -0.15583 | 1 | 173 |
| CT | GG | AA | GA | AG | GG | CC | GA | AA | -0.15178 | 1 | 2 |
| CC | GG | AC | GG | AG | GG | CT | GA | AA | -0.14425 | 1 | 1 |
| CT | GG | AA | GG | AA | GC | CC | GA | AA | -0.13891 | 1 | 2 |
| CT | GG | AA | GG | AG | GG | CC | GG | AA | -0.13739 | 1 | 28 |
| CT | GG | AA | GG | GG | GG | CC | GG | AA | -0.13739 | 1 | 1 |
| CT | GG | AA | GA | AG | GG | CC | GG | AA | -0.12459 | 1 | 11 |
| CT | GG | AA | GG | AG | GC | CC | GA | AA | -0.12047 | 1 | 3 |
| CT | GG | AA | GG | AG | GC | CC | AA | AA | -0.12047 | 1 | 1 |
| CT | GA | AA | GG | AA | GG | CC | GA | AA | -0.11183 | 1 | 2 |
| CT | GG | AA | GA | GG | GC | CC | GA | AA | -0.10768 | 1 | 1 |
| CC | GG | AC | GG | AA | GG | CC | GA | AA | -0.09586 | 1 | 60 |
| CC | GG | AA | GG | AA | GG | CT | GA | AA | -0.09402 | 1 | 46 |
| CC | GG | AA | GG | AA | GG | CT | AA | AA | -0.09402 | 1 | 18 |
| CT | GA | AA | GG | AA | GG | CC | GG | AA | -0.08464 | 1 | 4 |
| CC | GG | AC | GG | AG | GG | CC | GA | AA | -0.07742 | 1 | 3 |
| CC | GG | AA | GG | AG | GG | CT | GA | AA | -0.07558 | 1 | 381 |
| CC | GG | AA | GG | AG | GG | CT | AA | AA | -0.07558 | 1 | 101 |
| CC | GG | AA | GG | AG | GG | TT | AA | AA | -0.07558 | 1 | 1 |
| CC | GG | AA | GG | GG | GG | CT | GA | AA | -0.07558 | 1 | 16 |
| CC | GG | AA | GG | GG | GG | CT | AA | AA | -0.07558 | 1 | 8 |
| CC | GG | AC | GG | AA | GG | CC | GG | AA | -0.06867 | 1 | 186 |
| CC | GG | AA | GG | AA | GG | CT | GG | AA | -0.06683 | 1 | 8 |
| CC | GG | AC | GA | AG | GG | CC | GA | AA | -0.06462 | 1 | 7 |
| CC | GG | AA | GA | AG | GG | CT | GA | AA | -0.06278 | 1 | 30 |
| CC | GG | AA | GA | AG | GG | CT | AA | AA | -0.06278 | 1 | 12 |
| CC | GG | AA | GA | GG | GG | CT | GA | AA | -0.06278 | 1 | 25 |
| CC | GG | AA | GA | GG | GG | CT | AA | AA | -0.06278 | 1 | 13 |
| CC | GG | AC | GA | AA | GG | CC | GG | AA | -0.05587 | 1 | 1 |
| CC | GG | AC | GG | AA | GC | CC | GA | AA | -0.05175 | 1 | 10 |
| CC | GG | AC | GG | AG | GG | CC | GG | AA | -0.05023 | 1 | 9 |
| CC | GG | AA | GG | AA | GC | CT | AA | AA | -0.04992 | 1 | 5 |
| CC | GG | AC | GA | AG | GG | CC | GG | AA | -0.03743 | 1 | 20 |
| CC | GG | AA | GA | AG | GG | CT | GG | AA | -0.03559 | 1 | 3 |
| CC | GG | AC | GG | AG | GC | CC | GA | AA | -0.03331 | 1 | 1 |
| CC | GG | AA | GG | AG | GC | CT | GA | AA | -0.03147 | 1 | 1 |
| CC | GG | AA | GG | AG | GC | CT | AA | AA | -0.03147 | 1 | 9 |
| CC | GG | AA | GG | AA | GG | CC | GA | AA | -0.02719 | 1 | 20501 |
| CC | GG | AA | GG | AA | GG | CC | AA | AA | -0.02719 | 1 | 2655 |
| CC | GA | AC | GG | AA | GG | CC | GA | AA | -0.02467 | 1 | 2 |
| CC | GA | AA | GG | AA | GG | CT | GA | AA | -0.02283 | 1 | 3 |
| CC | GA | AA | GG | AA | GG | CT | AA | AA | -0.02283 | 1 | 1 |
| CC | GG | AC | GA | AG | GC | CC | GA | AA | -0.02051 | 1 | 2 |
| CC | GG | AA | GA | AG | GC | CT | AA | AA | -0.01868 | 1 | 1 |
| CC | GG | AA | GA | GG | GC | CT | AA | AA | -0.01868 | 1 | 2 |
| CC | GG | AA | GA | AA | GG | CC | GA | AA | -0.0144 | 1 | 3 |
| CC | GG | AA | GG | AG | GG | CC | GA | AA | -0.00875 | 1 | 3046 |
| CC | GG | AA | GG | AG | GG | CC | AA | AA | -0.00875 | 1 | 464 |
| CC | GG | AA | GG | GG | GG | CC | GA | AA | -0.00875 | 1 | 113 |
| CC | GG | AA | GG | GG | GG | CC | AA | AA | -0.00875 | 1 | 17 |
| CC | GA | AA | GG | AG | GG | CT | GA | AA | -0.00439 | 1 | 11 |
| CC | GA | AA | GG | AG | GG | CT | AA | AA | -0.00439 | 1 | 8 |
| CC | GG | AA | GG | AA | GG | CC | GG | AA | 0 | 2 | 38175 |
| CC | GA | AC | GG | AA | GG | CC | GG | AA | 0.002521 | 3 | 7 |
| CC | GG | AA | GA | AG | GG | CC | GA | AA | 0.004046 | 3 | 2834 |
| CC | GG | AA | GA | AG | GG | CC | AA | AA | 0.004046 | 3 | 417 |
| CC | GG | AA | GA | GG | GG | CC | GA | AA | 0.004046 | 3 | 211 |
| CC | GG | AA | GA | GG | GG | CC | AA | AA | 0.004046 | 3 | 42 |
| CC | GG | AA | AA | AG | GG | CC | GA | AA | 0.004046 | 3 | 1 |
| CC | GG | AA | AA | GG | GG | CC | GA | AA | 0.004046 | 3 | 71 |
| CC | GG | AA | AA | GG | GG | CC | AA | AA | 0.004046 | 3 | 11 |
| CC | GA | AA | GG | AA | GG | CT | GG | AA | 0.00436 | 3 | 1 |
| CC | GA | AA | GA | AG | GG | CT | GA | AA | 0.008406 | 3 | 1 |
| CC | GG | AA | GA | AA | GG | CC | GG | AA | 0.012798 | 3 | 4 |
| CC | GG | AA | GG | AA | GC | CC | GA | AA | 0.016914 | 3 | 2222 |
| CC | GG | AA | GG | AA | GC | CC | AA | AA | 0.016914 | 3 | 532 |
| CC | GG | AA | GG | AA | CC | CC | GA | AA | 0.016914 | 3 | 2 |
| CC | GG | AA | GG | AA | CC | CC | AA | AA | 0.016914 | 3 | 33 |
| CC | GG | AA | GG | AG | GG | CC | GG | AA | 0.018442 | 3 | 4355 |
| CC | GG | AA | GG | GG | GG | CC | GG | AA | 0.018442 | 3 | 109 |
| CC | GG | AA | GA | AA | GC | CC | GA | AA | 0.029712 | 3 | 1 |
| CC | GG | AA | GA | AG | GG | CC | GG | AA | 0.031239 | 3 | 5034 |
| CC | GG | AA | GA | GG | GG | CC | GG | AA | 0.031239 | 3 | 258 |
| CC | GG | AA | AA | AG | GG | CC | GG | AA | 0.031239 | 3 | 7 |
| CC | GG | AA | AA | GG | GG | CC | GG | AA | 0.031239 | 3 | 149 |
| CC | GG | AA | GG | AG | GC | CC | GA | AA | 0.035356 | 3 | 665 |
| CC | GG | AA | GG | AG | GC | CC | AA | AA | 0.035356 | 3 | 178 |
| CC | GG | AA | GG | AG | CC | CC | GA | AA | 0.035356 | 3 | 6 |
| CC | GG | AA | GG | AG | CC | CC | AA | AA | 0.035356 | 3 | 21 |
| CC | GG | AA | GG | GG | GC | CC | GA | AA | 0.035356 | 3 | 21 |
| CC | GG | AA | GG | GG | GC | CC | AA | AA | 0.035356 | 3 | 12 |
| CC | GG | AA | GG | GG | CC | CC | AA | AA | 0.035356 | 3 | 1 |
| CC | GA | AA | GG | AG | GC | CT | AA | AA | 0.039716 | 3 | 2 |
| CC | GA | AA | GG | AA | GG | CC | GA | AA | 0.043996 | 3 | 1695 |
| CC | GA | AA | GG | AA | GG | CC | AA | AA | 0.043996 | 3 | 230 |
| CC | AA | AA | GG | AA | GG | CC | GA | AA | 0.043996 | 3 | 29 |
| CC | AA | AA | GG | AA | GG | CC | AA | AA | 0.043996 | 3 | 7 |
| CC | GG | AA | GG | AA | GC | CC | GG | AA | 0.044108 | 3 | 62 |
| CC | GG | AA | GA | AG | GC | CC | GA | AA | 0.048154 | 3 | 375 |
| CC | GG | AA | GA | AG | GC | CC | AA | AA | 0.048154 | 3 | 84 |
| CC | GG | AA | GA | AG | CC | CC | AA | AA | 0.048154 | 3 | 5 |
| CC | GG | AA | GA | GG | GC | CC | GA | AA | 0.048154 | 3 | 47 |
| CC | GG | AA | GA | GG | GC | CC | AA | AA | 0.048154 | 3 | 13 |
| CC | GG | AA | GA | GG | CC | CC | AA | AA | 0.048154 | 3 | 3 |
| CC | GG | AA | AA | GG | GC | CC | GA | AA | 0.048154 | 3 | 14 |
| CC | GG | AA | AA | GG | GC | CC | AA | AA | 0.048154 | 3 | 8 |
| CC | GA | AA | GG | AG | GG | CC | GA | AA | 0.062438 | 3 | 125 |
| CC | GA | AA | GG | AG | GG | CC | AA | AA | 0.062438 | 3 | 25 |
| CC | GG | AA | GG | AG | GC | CC | GG | AA | 0.062549 | 3 | 121 |
| CC | GG | AA | GG | AG | CC | CC | GG | AA | 0.062549 | 3 | 1 |
| CC | GG | AA | GG | GG | GC | CC | GG | AA | 0.062549 | 3 | 7 |
| CC | GA | AA | GG | AA | GG | CC | GG | AA | 0.07119 | 3 | 2773 |
| CC | AA | AA | GG | AA | GG | CC | GG | AA | 0.07119 | 3 | 58 |
| CC | GA | AA | GA | AG | GG | CC | GA | AA | 0.075236 | 3 | 118 |
| CC | GA | AA | GA | AG | GG | CC | AA | AA | 0.075236 | 3 | 21 |
| CC | GG | AA | GA | AG | GC | CC | GG | AA | 0.075347 | 3 | 5 |
| CC | GG | AA | GA | GG | GC | CC | GG | AA | 0.075347 | 3 | 5 |
| CC | GA | AA | GG | AA | GC | CC | GA | AA | 0.088104 | 3 | 286 |
| CC | GA | AA | GG | AA | GC | CC | AA | AA | 0.088104 | 3 | 68 |
| CC | GA | AA | GG | AA | CC | CC | AA | AA | 0.088104 | 3 | 3 |
| CC | AA | AA | GG | AA | GC | CC | GA | AA | 0.088104 | 3 | 7 |
| CC | AA | AA | GG | AA | GC | CC | AA | AA | 0.088104 | 3 | 3 |
| CC | GA | AA | GG | AG | GG | CC | GG | AA | 0.089631 | 3 | 170 |
| CC | GA | AA | GA | AG | GG | CC | GG | AA | 0.102429 | 3 | 202 |
| CC | GA | AA | GA | GG | GG | CC | GG | AA | 0.102429 | 3 | 1 |
| CC | GA | AA | GG | AG | GC | CC | GA | AA | 0.106545 | 3 | 33 |
| CC | GA | AA | GG | AG | GC | CC | AA | AA | 0.106545 | 3 | 8 |
| CC | GA | AA | GG | AG | CC | CC | GA | AA | 0.106545 | 3 | 1 |
| CC | GA | AA | GG | AG | CC | CC | AA | AA | 0.106545 | 3 | 2 |
| CC | GA | AA | GG | AA | GC | CC | GG | AA | 0.115297 | 3 | 5 |
| CC | GA | AA | GA | AG | GC | CC | GA | AA | 0.119343 | 3 | 19 |
| CC | GA | AA | GA | AG | GC | CC | AA | AA | 0.119343 | 3 | 9 |
| CC | GA | AA | GA | GG | GC | CC | GA | AA | 0.119343 | 3 | 1 |
| CC | GA | AA | GG | AG | GC | CC | GG | AA | 0.133739 | 3 | 4 |

For all existing genotype combinations their single allele weights were summarized into a weighted allele score. Minor alleles are marked in red.

# Supplementary Table 2 Explained risk in the association from genetic *ABCA1* variation based on 90,556 individuals from the Copenhagen General Population Study and the Copenhagen City Heart Study.

|  | Number of events | Via higher HDL cholesterol  Percent (95%CI) |
| --- | --- | --- |
| All age-related macular degeneration | 1,512 | 6 (3-14) |
| Nonneovascular age-related macular degeneration | 874 | 8 (3-30) |
| Neovascular age-related macular degeneration | 1,064 | 6 (3-17) |

Adjusted for age, sex, and cohort. Results are per one higher weighted allele score tertile.

**
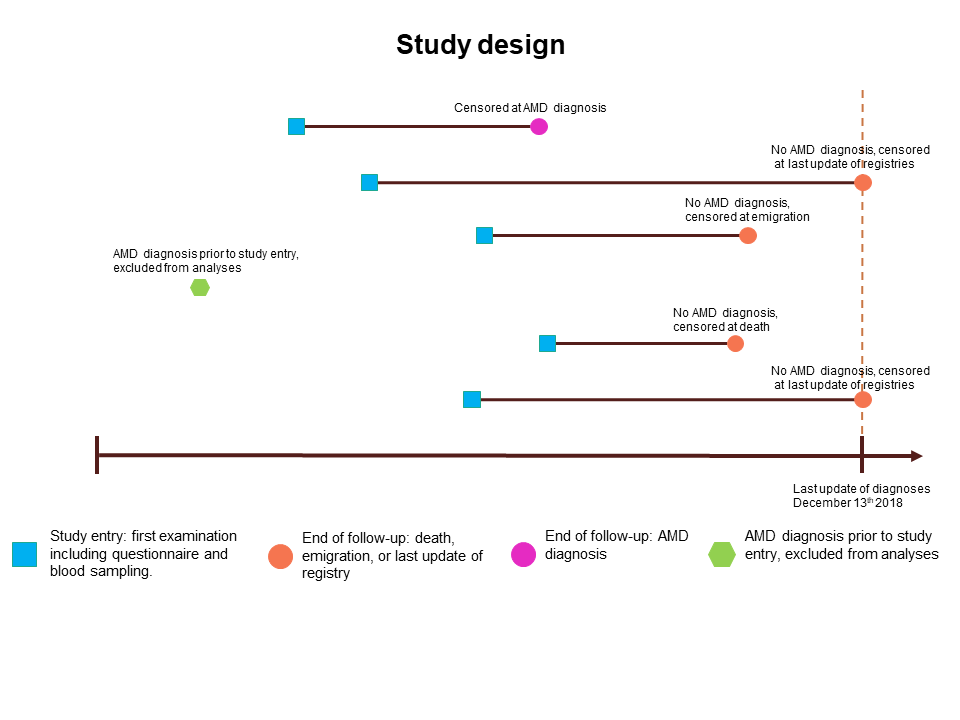
****Supplementary Figure 1 Study design**.

Participants entered the study at their first examination. AMD diagnoses before study entry were not included in analyses. End of follow-up was at AMD diagnosis, death, emigration, or last update of registry. AMD = age-related macular degeneration.


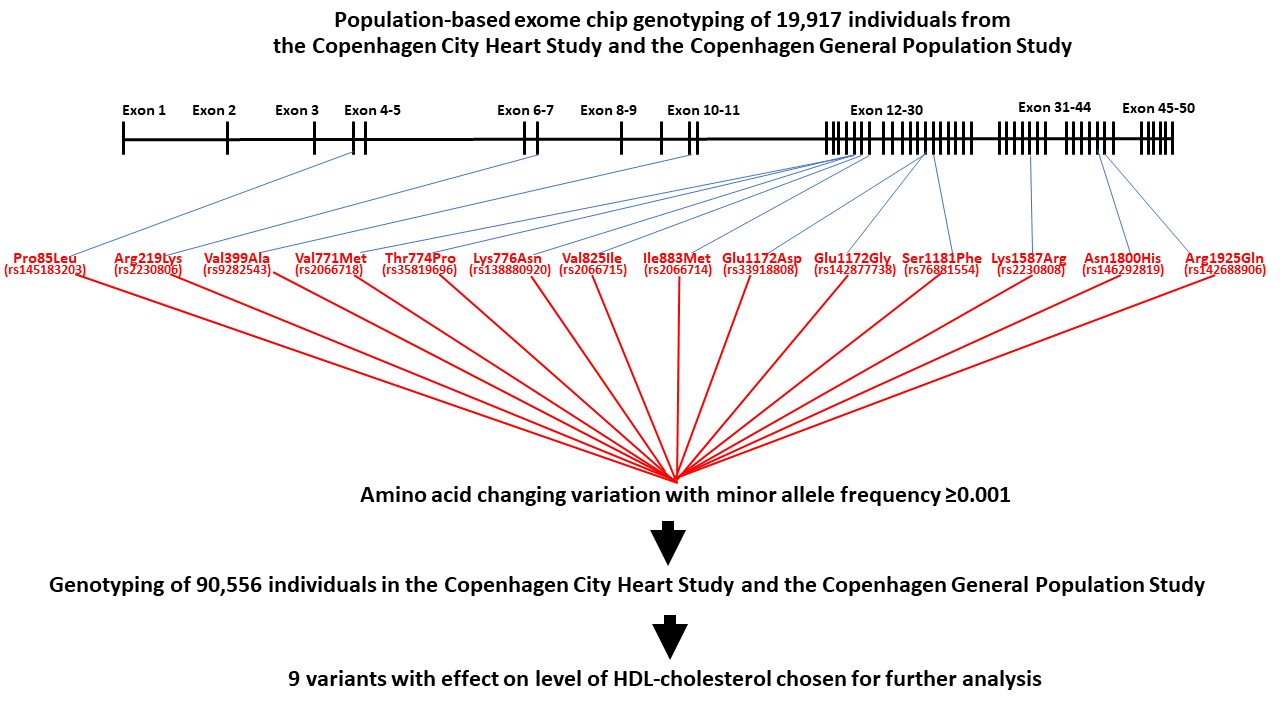


# Supplementary Figure 2 Genotyping of *ABCA1* amino acid changing variants in the CCHS and CGPS.

Based on data from an exome chip all amino acid changing genetic variants with a minor allele frequency above 0.001 in the *ABCA1* gene were chosen for genotyping in the CCHS and CGPS. The nine genetic variants that were associated with HDL cholesterol concentrations in CGPS and CCHS were chosen for further statistical analysis. ABCA1 = adenosine triphosphate-binding cassette transporter A1; CCHS = Copenhagen City Heart Study; CGPS = Copenhagen General Population Study.

#
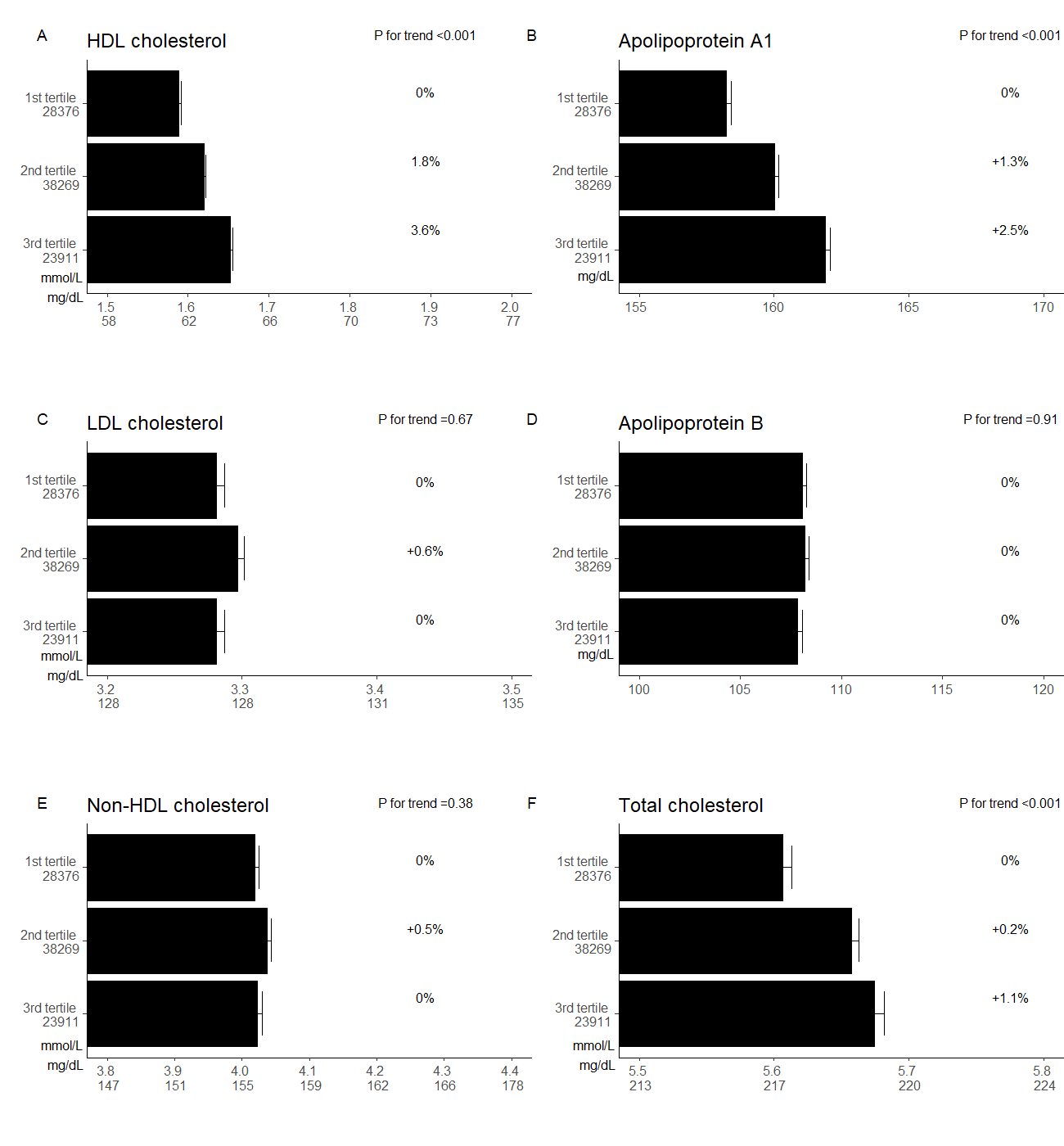
Supplementary Figure 3 *ABCA1* weighted allele score in tertiles and lipid, lipoprotein, and apolipoprotein concentrations.

Concentrations of HDL cholesterol (panel A), apolipoprotein A1 (panel B), LDL cholesterol (panel C), apolipoprotein B (panel D), non-HDL cholesterol (panel E), and total cholesterol (panel F) as a function of amino acid changing *ABCA1* weighted allele score tertiles. SI conversion factors: To convert cholesterol to millimoles per liter, multiply by 0.0259; triglycerides to millimoles per liter, multiply by 0.0113. ABCA1 = adenosine triphosphate-binding cassette transporter A1; HDL = high-density lipoprotein. LDL = low-density lipoprotein.

#
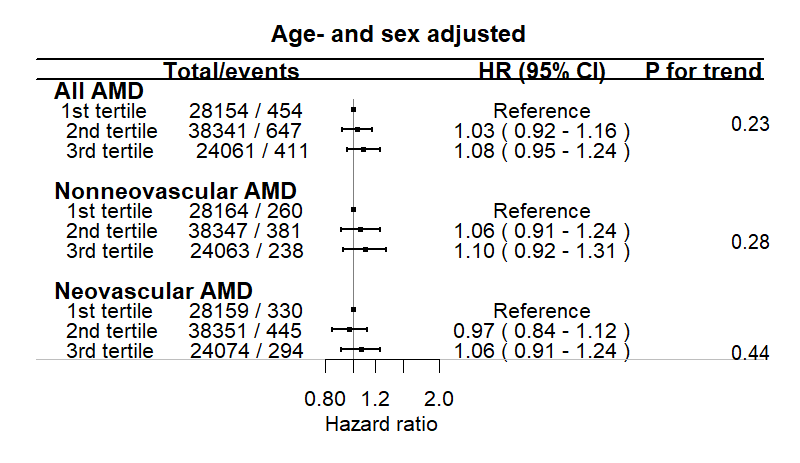
**Supplementary Figure 4 Risk of age-related macular degeneration as a function of *ABCA1* weighted allele score in tertiles using external weights**

Hazard ratios (HRs) and 95% confidence intervals are from Cox regression models. The allele score was weighted on the effect on plasma HDL cholesterol obtained from UK Biobank via https://app.genebass.org/. Beta-coeficients were only available for 8 out of 9 variants. Adjustment was for age, sex, and cohort. ABCA1 = adenosine triphosphate-binding cassette transporter A1; AMD = age-related macular degeneration; CCHS = Copenhagen City Heart Study; CGPS = Copenhagen General Population Study; CI=confidence interval; HDL = high-density lipoprotein.

#
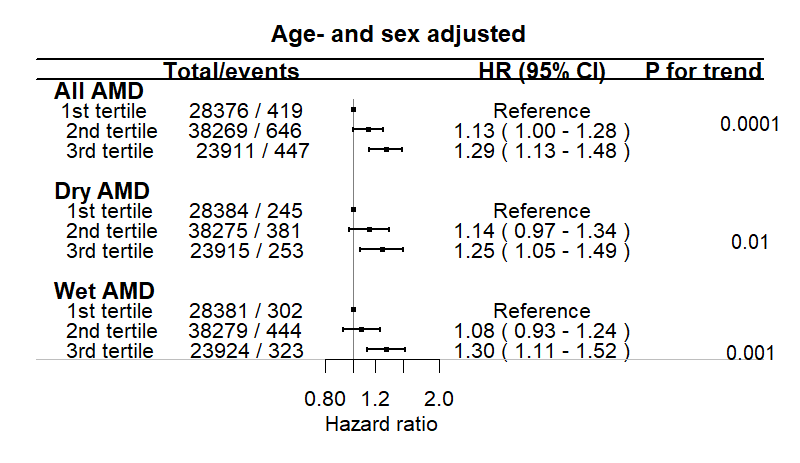
Supplementary Figure 5 **Risk of age-related macular degeneration as a function of *ABCA1* weighted allele score in tertiles multivariable adjusted including LDL and total cholesterol.**

Hazard ratios (HRs) and 95% confidence intervals are from Cox regression models. The allele score was weighted on the effect on plasma HDL cholesterol obtained from the Copenhagen General Population Study and the Copenhagen City Heart Study. Adjustment was for age, sex, cohort, body mass index, hypertension, diabetes mellitus, smoking, alcohol consumption, physical inactivity, menopausal status, and hormonal replacement therapy (only women), lipid-lowering therapy, education, LDL cholesterol, and total cholesterol. ABCA1 = adenosine triphosphate-binding cassette transporter A1; AMD = age-related macular degeneration; CI=confidence interval; HDL = high-density lipoprotein.


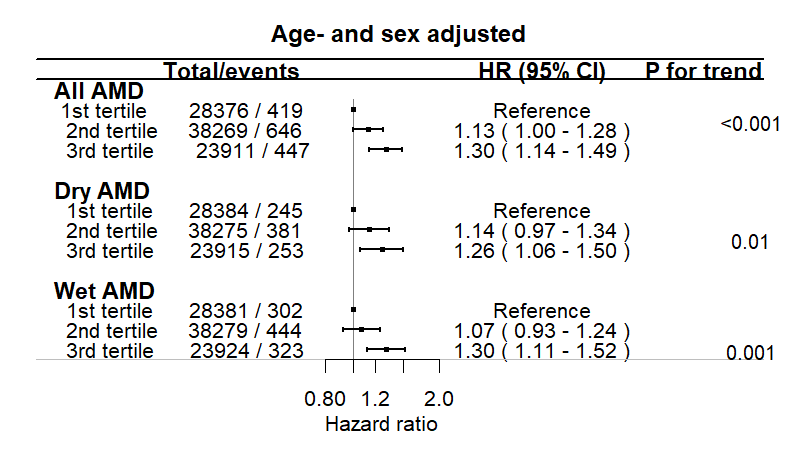
Supplementary Figure 6 Risk of age-related macular degeneration as a function of *ABCA1* weighted allele score in tertiles multivariable adjusted including skin cancer.

Hazard ratios (HRs) and 95% confidence intervals are from Cox regression models. The allele score was weighted on the effect on plasma HDL cholesterol obtained from the Copenhagen General Population Study and the Copenhagen City Heart Study. Adjustment was for age, sex, cohort, body mass index, hypertension, diabetes mellitus, smoking, alcohol consumption, physical inactivity, menopausal status, and hormonal replacement therapy (only women), lipid-lowering therapy, education, and skin cancer. ABCA1 = adenosine triphosphate-binding cassette transporter A1; AMD = age-related macular degeneration; CI=confidence interval; HDL = high-density lipoprotein.

#
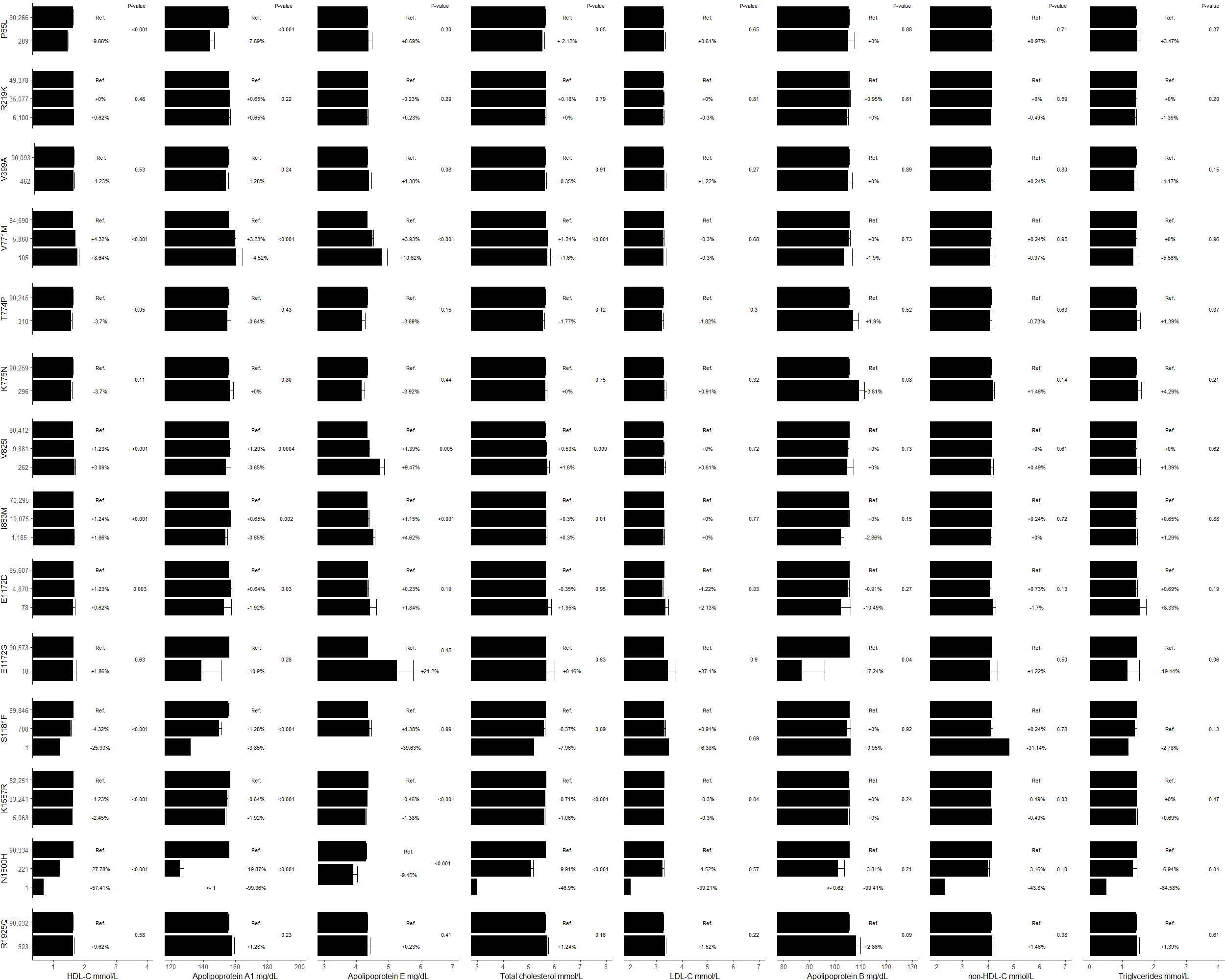


# Supplementary Figure 7 *ABCA1* variants and lipid, lipoprotein, and apolipoprotein levels.

Levels of HDL cholesterol, apolipoprotein A1, apolipoprotein E, total cholesterol, LDL cholesterol, apolipoprotein B, non-HDL cholesterol, and triglycerides as a function of *ABCA1* genotype. SI conversion factors: To convert cholesterol to millimoles per liter, multiply by 0.0259; triglycerides to millimoles per liter, multiply by 0.0113. ABCA1 = adenosine triphosphate-binding cassette transporter A1; HDL = high-density lipoprotein; C = cholesterol; HDL = high-density lipoprotein; LDL = low-density lipoprotein.

#
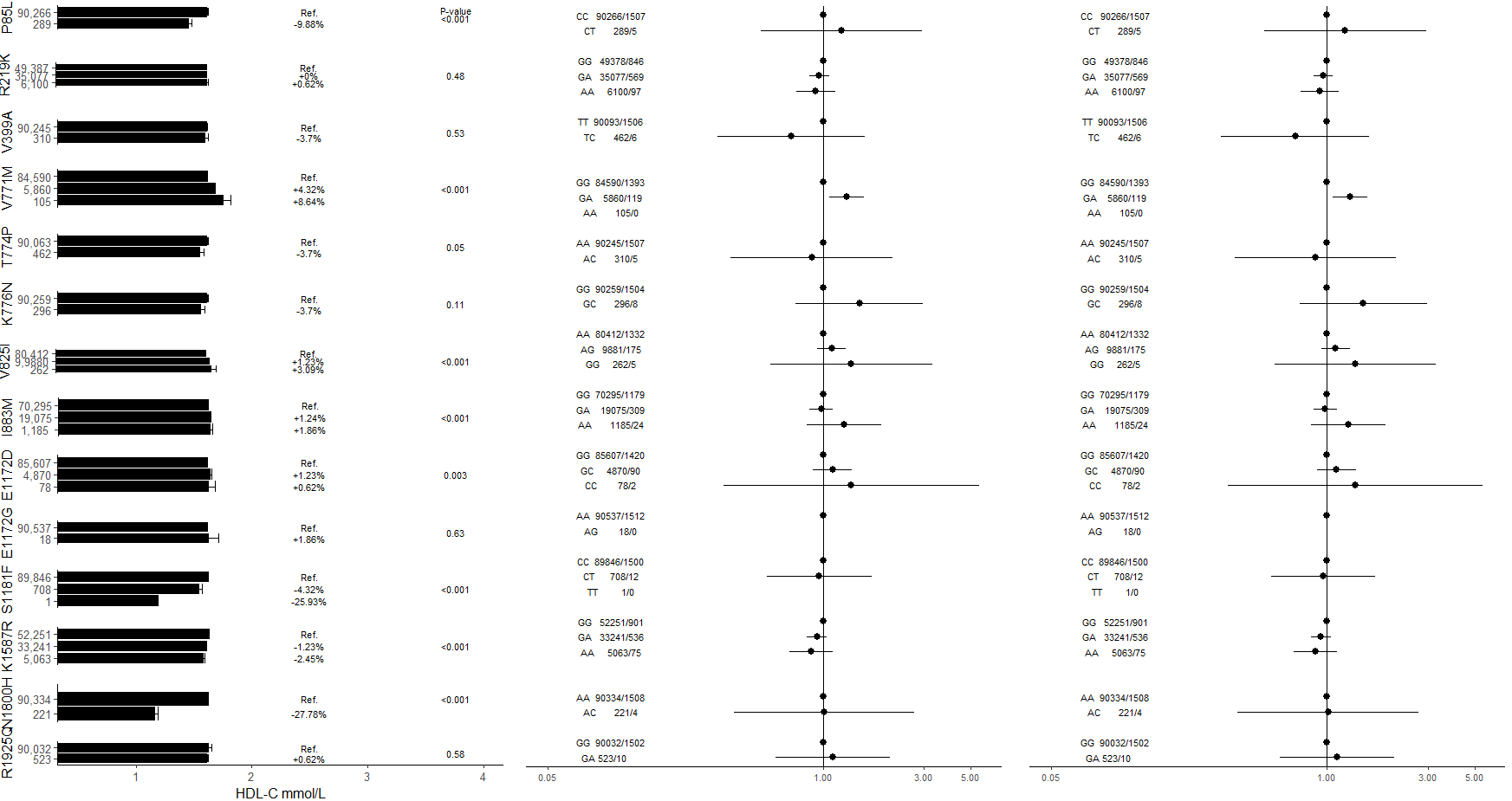
Supplementary Figure 8 Concentrations of HDL cholesterol and risk of AMD as a function of *ABCA1* genotype.

Hazard ratios (HRs) and 95% confidence intervals are from Cox regression models. Adjustment was for age, sex, and cohort (middle panel), and multivariable for age, sex, cohort, body mass index, hypertension, diabetes mellitus, smoking, alcohol consumption, physical inactivity, menopausal status, and hormonal replacement therapy (only women), lipid-lowering therapy, and education (left panel). SI conversion factors: To convert cholesterol to millimoles per liter, multiply by 0.0259. CI=confidence interval. HDL = high-density lipoprotein.


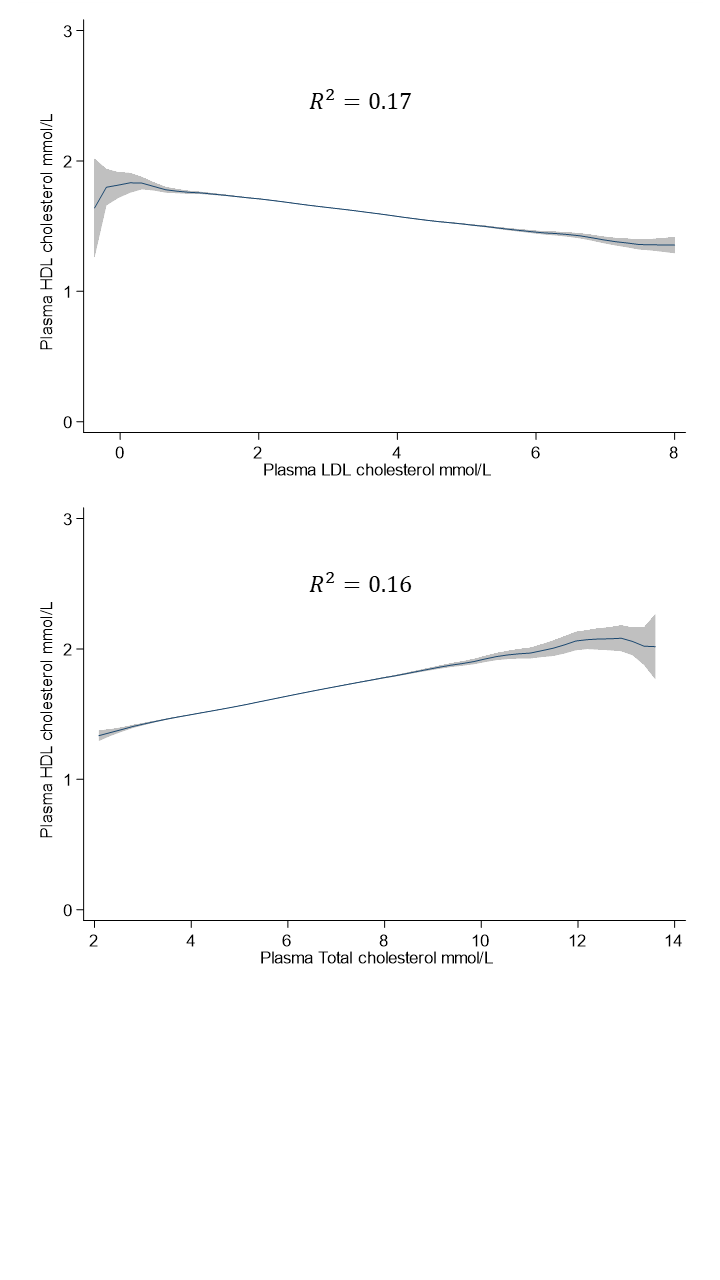


# Supplementary Figure 9 Correlation between plasma HDL cholesterol, LDL cholesterol, and total cholesterol

Association of plasma HDL cholesterol with LDL- and total cholesterol. Based on the Copenhagen General Population Study and the Copenhagen City Heart Study. Dark blue line are smoothed polynomial functions and 95% confidence interval (CI) indicated with light grey area, obtained from multiple linear regression analysis. HDL = high-density lipoprotein; LDL = low-density lipoprotein.

#
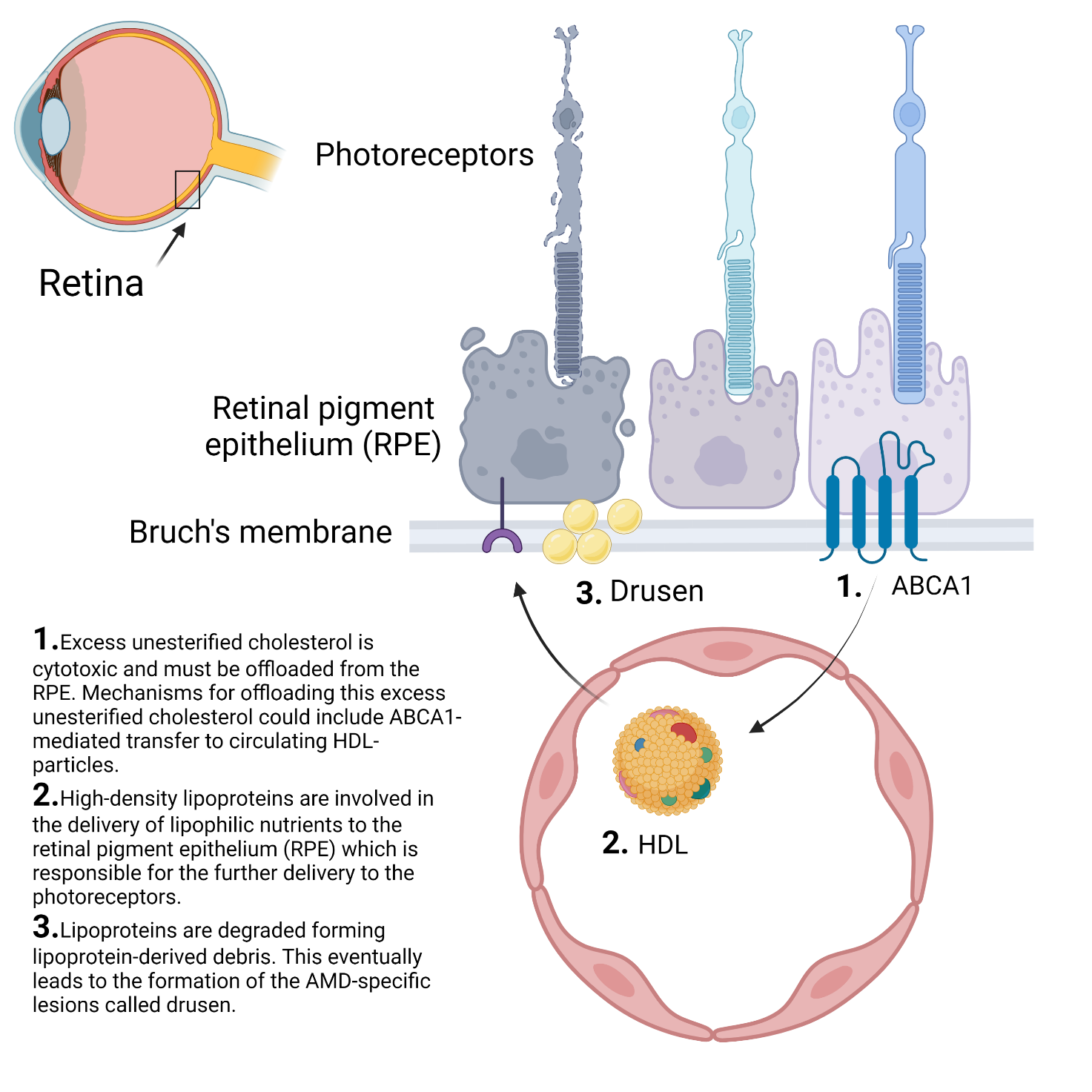
Supplementary Figure 10 The Oil Spill Hypothesis in Bruch’s membrane.

ABCA1 = adenosine triphosphate-binding cassette transporter A1; HDL = high-density lipoprotein. Modified from Curcio et al., Br J Ophtalmol 2011; 1-8. Created with Biorender.com.
